# Contrasting academic approaches to COVID-19 vaccine production and distribution: What can the Oxford and Texas experiences teach us about pandemic response?

**DOI:** 10.1093/haschl/qxae012

**Published:** 2024-01-31

**Authors:** Jorge L Contreras, Kenneth C Shadlen

**Affiliations:** University of Utah S.J. Quinney College of Law, Salt Lake City, UT84112, United States; Department of Human Genetics, University of Utah School of Medicine, Salt Lake City, UT 84112, United States; Department of International Development, London School of Economics and Political Science, London WC2A 2AE, United Kingdom

**Keywords:** technology transfer, innovation, production, open source licensing, pandemic response, vaccine, COVID-19

## Abstract

This article contrasts the different approaches to COVID-19 vaccine development adopted by Oxford University, on one hand, and Texas Children's Hospital and Baylor College of Medicine (collectively, Texas), on the other hand. Texas was praised widely in the press and academic literature for adopting an “open source” approach to vaccine development. Oxford, however, chose to license its vaccine technology to pharmaceutical manufacturer AstraZeneca and received significant public criticism as a result. Yet the Oxford vaccine reached far more individuals in developing countries than the Texas vaccine. We compare the two vaccines' experiences, drawing attention to a constellation of interrelated elements that contribute to a successful vaccine production program, including not only IP licensing, but also timing, technology transfer, and resource mobilization, all in the context of the prevailing funding environments. This comparative analysis sheds light on how the innovation ecosystem functioned during the COVID-19 pandemic, providing useful insights for policy makers and advocates as they prepare for future pandemics and other global health challenges.

## Introduction

In December 2021, approximately 2 years into the COVID-19 pandemic, researchers at Baylor College of Medicine and Texas Children's Hospital announced a new COVID-19 vaccine that was authorized for emergency use in India. Unlike the vaccines developed by Moderna, BioNTech, and other private firms (which were also often based on early academic research), the Texas vaccine would not be patented, and instead would be made available for modest fees to manufacturers throughout the developing world.^1,2^ This “open source” approach to vaccine development attracted widespread praise and resulted in its leaders—Drs. Peter Hotez and Maria Elena Bottazzi—being nominated for the Nobel Peace Prize.^3^

The positive public reaction to the open-source Texas vaccine can be contrasted with the more skeptical public reaction to the vaccine development strategy pursued by Oxford University. Oxford was initially applauded for its commitment to offer nonexclusive, royalty-free licenses of its COVID-19 technologies, but attracted criticism when, in April 2020, it instead granted a royalty-bearing, exclusive license to the global pharmaceutical firm AstraZeneca.^4-8^ Some academic commentators lumped Oxford together with commercial vaccine manufacturers in exhibiting a “protectionist approach to IP” that limited “the volume and regional spread of production capacity of COVID-19 vaccines, endangering global health efforts,”^9^ while others accused Oxford of not sharing the benefits of publicly funded research “fairly and equitably with the global population.”^7^ These critiques resonate with broader and longstanding public sentiments that publicly funded research institutions have failed to live up to their public missions by prioritizing the pursuit of commercial gains.^10-13^

But, despite the differing public responses to the Texas and Oxford vaccine strategies, their respective results on the ground call for a reassessment. Table 1 compares these 2 approaches along several dimensions, along with 2 of the more prominent commercial vaccine efforts. The Texas vaccine, produced primarily by 1 partner in India and another in Indonesia, has had a total output to date of approximately 100 million doses. By contrast, the Oxford vaccine, produced by a global network of manufacturers, coordinated by AstraZeneca, has had a combined output in excess of 3 billion doses, mostly distributed at low cost in poorer countries. While the Texas effort was lauded for its public spirit, the Oxford vaccine had a greater positive impact on global health, particularly in the developing world.

**Table 1.** Comparison of COVID-19 vaccine developer strategies and outcomes.

	Academic		Industry	
	Oxford	Texas	Moderna	BioNTech
Technology	Viral vector	Protein	mRNA	mRNA
Patented	Yes	No	Yes	Yes
Global development and commercialization partner	AstraZeneca	None	None	Pfizer
Technology transfer	AstraZeneca to manufacturing partners	Direct to manufacturing partners	Direct to manufacturing partners	Direct to manufacturing partners
Manufacturing^a^	Global manufacturing network (12 countries)	Licensed Manufacturing partners (2 countries)	In-house + manufacturing partners (3 countries)	In-house + Pfizer (2 countries)
Doses manufactured^b^	3096 million	100 million	1163 million	3687 million
Adult authorizations and approvals^c^	168 offices plus WHO EUL/PQ	4 offices^d^	95 offices plus WHO EUL/PQ	131 offices plus WHO EUL/PQ
Date of first authorization^c^	December 30, 2020	December 28, 2021	December 17, 2020	December 2, 2020
Pricing/distribution constraints	Commitment to distribute at cost in poor countries	None disclosed	None disclosed	None disclosed

Abbreviations: EUL/PQ, Emergency Use Listing/Prequalification; WHO, World Health Organization.

^a^Drug substance only (ie, excluding fill-finish production). Source: Airfinity.

^b^As of June 2023. Source: Airfinity.

^c^Source: Airfinity and WHO.

^d^In January 2024 (as this article was being published), the Texas vaccine received EUL/PQ from the WHO.

In this Policy Inquiry we analyze and compare the trajectories of these 2 university-based projects. We do so without seeking to compare the efficacy of the vaccines, which were tested and approved under different regulatory regimes, making comparison difficult. In our estimation, both vaccines were largely viewed as effective. One measurable factor contributing to the differences in the results of the 2 vaccine projects was timing. The Oxford-AstraZeneca vaccine was first authorized for use (in the United Kingdom) on December 30, 2020, and subsequently approved in more than 100 countries by the end of March 2021. In contrast, the Texas vaccine did not receive its first regulatory approval (in India) until December 28, 2021, a full year after Oxford-AstraZeneca—an eternity in terms of pandemic response. On this basis alone, it is not surprising that more doses of the Oxford-AstraZeneca vaccine were manufactured and administered around the world.

But there were more factors at work than timing alone. An important reason for the difference in production of the 2 vaccines arises from the extent of technology transfer, which should not be considered independently from the institutions’ licensing approaches. Specifically, we consider the role played by Oxford's partnership with AstraZeneca. By digging more deeply into the Oxford–AstraZeneca relationship we observe that this vaccine's contributions to the global vaccination campaign derive from licensing and production strategies that distinguish it from both open-source and traditional commercial approaches. We also point to the importance of studying distinct licensing approaches in the context of the funding environments and ecosystems in which they operate.

Below, we first present a brief overview of the role of patents in the development of biomedical products. We then compare the technology transfer approaches of Oxford-AstraZeneca and Texas. Finally, we look in more detail at the Oxford partnership with AstraZeneca, and the implications of the comparative analysis for the COVID-19 innovation ecosystem.

## Patents

Patents give their owners exclusive rights to exploit claimed inventions in the countries where the patents are issued. In accordance with the World Trade Organization's (WTO) Agreement on Trade Related Aspects of Intellectual Property (TRIPS), most countries extend patent protection to biological and pharmaceutical products. With respect to biomedical products, such as drugs, diagnostics, and vaccines, patents typically have played important roles in establishing market exclusivity for their owners, enabling them to operate without competition for the period that the patents are in effect and thus charge any price that the market will bear. As a result, these firms can both recoup significant development costs and also earn sizeable profits. Yet, for this reason, patented pharmaceutical products are often beyond the reach of patients in poor countries, where government health budgets are more constrained and large shares of the population often pay out-of-pocket for their medicines.^14^

It is not surprising, then, that the prospect of patents on COVID-19 diagnostics, vaccines, and therapeutics gave rise to concerns from the early days of the pandemic. In response, public and private initiatives were launched to facilitate institutions and firms making their patents and technologies broadly available,^15,16^ and in late 2020 the WTO began to consider a proposed waiver of its member states’ obligations regarding COVID-related intellectual property (IP).^17^

As noted above, the Texas researchers did not patent their vaccine, meaning that any entity could legally produce it anywhere in the world without risk of infringing on their patents. Oxford, by contrast, which well before the pandemic had constructed a patent portfolio on viral vector technologies managed by its spin-out firm (Vaccietch) and the university's technology transfer agent (Oxford University Innovation), licensed its patents on an exclusive basis to AstraZeneca.^6,18^ Legally, this means that no other entity could make, use, or sell the Oxford vaccine in any country where a patent was issued, without the permission of Oxford and AstraZeneca. At first glance, then, the Oxford strategy resembles that of commercial ventures such as BioNTech, which partnered (at an even earlier stage) with Pfizer to develop and manufacture its mRNA vaccine^19^ (see Table 1). Yet, as we discuss below, this did not turn out to be the case.

## Technology transfer, partnerships, and global production

The ability of pharmaceutical firms to produce vaccines at the scale and speed required by pandemic conditions depends on more than the product's patent protection. Access to know-how regarding complex manufacturing, analytic, and quality-control processes is also essential.^20-22^ This, in turn, calls for technology transfer: sharing of the full package of trade secrets, data, and know-how that enables regulatory approval, manufacture, and distribution of a final product. Importantly, not all of the know-how that potential partners need is written (often referred to as “codified”), and transferring noncodified, “tacit” knowledge typically requires direct engagement between originators and the recipients of this knowledge.

Technology transfer, in addition to being essential, is also resource-intensive.^23^ It has costs in terms of, among other things, identifying partners with appropriate capabilities, helping partners adapt their facilities, teaching the essential steps of production and quality control, and engaging during the course of manufacturing. And given the importance of transferring noncodified knowledge, there are human resource constraints too. To the extent that more recipients, with diverse capabilities and therefore diverse needs, are involved, the requirements on the transferor of technology multiply.^24^

Both the Texas and Oxford vaccines were the objects of technology transfer, but to different degrees. Despite the Texas group's identification of its non-patenting strategy as open source, the group did not make manufacturing know-how broadly available to the public, as a developer of open-source software might,^25^ but only transferred this information to selected industrial partners in low-income countries. Specifically, the Texas group made a package of testing and design data, as well as ongoing assistance, available to manufacturers in a handful of low-income countries that agreed to abide by specified licensing conditions, including the payment of monetary royalties. Thus, unlike open-source software, which is typically released free of charge in a format that can be used by any programmer familiar with the relevant programming language, sharing the know-how required for vaccine production also required more hands-on guidance and interaction. The Texas approach yielded 2 partnerships of which we are aware: with Biological E in India and Bio Farma in Indonesia, both important global producers that are members of the Developing Countries Vaccine Manufacturers Network.^26^

Oxford, in contrast, relied on AstraZeneca, which transferred technology simultaneously to manufacturing partners in multiple countries.^27^ The most important partner in terms of output was the Serum Institute of India, the world's largest vaccine producer (by volume), and which alone ended up accounting for approximately 60% of doses produced. In addition the arrangements with the Serum Institute, AstraZeneca built a global manufacturing network for the vaccine. That is, the Oxford network ended up with 2 types of partners: AstraZeneca as principal licensee, and manufacturers around the globe that participated as sublicensees and contract manufacturers. To be sure, not all of Oxford-AstraZeneca's partnerships were equally successful; some of the partners in the distributed manufacturing network ended up producing comparatively little vaccine.^28^ (The reliance on the Serum Institute as the most important source of global supply became problematic in 2021 when the Indian government imposed an export ban.) Yet, the commitment to transfer technology globally stands out: AstraZeneca's engineers shared technology and know-how for drug substance production with 12 partners in Asia (China, India, South Korea, Thailand, Japan) and Latin America (Argentina, Brazil), as well as partners in Australia, Belgium, Netherlands, the United Kingdom, and the United States; and multiple additional partners (eg, in Mexico) were trained to execute the final “fill-finish” stages—in sum, 25 different manufacturers in 15 countries.^28-30^

If anything, widespread technology transfer of this sort should have been more difficult for the Oxford vaccine than for the Texas vaccine, as Oxford's viral vector approach was at the cutting edge of “digital” genetic vaccines^31^ that calls on novel manufacturing processes, while the Texas vaccine's protein-based approach is common throughout the Global South, based on production processes and facilities widely in use.^28,32-34^ The challenges of transferring technology to bring partners up to speed were almost certainly greater in the case of the Oxford vaccine (though it is worth noting that early in the vaccine development process, before partnering with AstraZeneca, Oxford scientists innovated to simplify the production process in ways that would make more technology transfer possible than otherwise may have been the case^35^). Yet, the differences in the size of the different vaccines’ manufacturing partnerships and production output are notable.

The differences in the Texas and Oxford vaccines’ outcomes, both the size of the production networks and the volumes of output generated, remind us that technology transfer entails more than removing fear of litigation and sharing proprietary data. At least during a pandemic, when speed is of the essence, technology transfer also entails hands-on originator engagement to share noncodified know-how—that is, tacit knowledge. To be sure, the Texas team did all of this—they removed fear of patent litigation, they made their data and information available, and they offered hands-on support to aid their partners in manufacturing. Yet, acting alone, they were necessarily limited in how much they could contribute, and thus the size of the global network they could create.

## Licensing for global production and distribution

AstraZeneca, with little previous vaccine experience, was not the first pharmaceutical firm that Oxford approached.^35,36^ Yet, as it turned out, what mattered most was not vaccine manufacturing prowess per se, but rather the administrative, organizational, financial, managerial, and technological capabilities that a giant global pharmaceutical firm like AstraZeneca could apply to the project, including its experience in outward technology transfer.

AstraZeneca had the resources and administrative capacity to engage in the global identification of partners and to work hands-on with multiple partners, simultaneously, to help them absorb and use technology quickly. Importantly, as an experienced global manufacturer, AstraZeneca also was able to mobilize governments and philanthropic organizations to contribute essential resources to enable expanded investment in manufacturing. As the principal Oxford scientists have explained, after AstraZeneca came on board “things *really* took off. With their existing relationships with major manufacturing sites and the financing power to be able to commit to contracts, AstraZeneca was able to activate a programme of global production that was entirely beyond the scope of a UK university-led project.” pp. 145-146 The involvement of this third actor thus turned out to be important for finding global partners, triggering and accessing additional funding, and executing a program of global technology transfer.

Just as the Texas approach differed from traditional open-source software licensing, the Oxford licensing approach was not an off-the-shelf exclusive license. Rather, it appears to include critical public interest (global health-oriented) provisions.^37^ Oxford expected AstraZeneca to establish global partnerships to decentralize production, with some partners operating on a “no profit, no loss” basis. That is, the breadth of the ensuing technology transfer, supported by additional external funding to proceed “at risk” in 2020 while trials were in process, appears to have been part of Oxford's licensing strategy, as was Oxford's expectation that vaccine doses produced by AstraZeneca's global network be made available at affordable prices in developing countries.^6,28,36-38^ AstraZeneca would do so by selling at discounted prices directly to poorer countries and at-cost to the nonprofit COVAX organization, which was founded in 2020 to procure vaccines and make them available for distribution in 92 low- and middle-income countries.^39^ Of course, caution is warranted in making statements about the prices of vaccines during the pandemic, as these were opaque and inconsistent; South Africa, for example, reportedly paid more for the Oxford-AstraZeneca vaccine than EU countries did.^40^

Here it is worth returning to the issue of timing that was noted in the Introduction. By the time the Texas vaccine completed trials and was ready for production and distribution in late 2021, a year after the Oxford-AstraZeneca vaccine became available, the COVID-19 vaccine market was saturated, with multiple products based on different technological platforms available. Indeed, by the end of 2021, and in stark contrast to the first 2 years of the pandemic, the supply of vaccines was outstripping demand. In such a context there may be little reason to expect Texas even to have aspired to a global manufacturing network along the lines of that achieved by Oxford and AstraZeneca.^2^ Yet, the comparatively late arrival of the Texas vaccine, well after major variants such as Delta had run their course, is partially endogenous to other issues we have discussed. If the Texas vaccine had an external partner with global reach and benefitted from additional funding, it, too, might have advanced more quickly, being able to initiate technology transfer and clinical trial design at risk during the course of product development, as the Oxford-AstraZeneca vaccine (and the other externally supported candidates) did.

## Conclusion

In this article we offer a comparative view of the Texas and Oxford vaccines’ trajectories, focusing primarily on their approaches to IP licensing and technology transfer, their engagement with partner manufacturers, and the roles they ultimately played in global pandemic response.

The Texas open-source vaccine project was viewed as being responsive to widespread concerns during the COVID-19 pandemic about equitable access to vaccines and treatments, particularly in low- and middle-income countries. Building on traditions of open science and global public health, the Texas researchers brought their own track record in neglected tropical diseases to bear on the COVID-19 vaccine challenge. As such, they achieved success with the distribution of low-cost vaccines in India and Indonesia, and potentially in other countries where the vaccine is authorized for use.

Had more and larger external funders supported the Texas vaccine, the project might have developed faster and been accompanied by earlier and more expansive technology transfer and production. However, in a world where funders were directing resources to known partners that they expected to achieve big impacts quickly, Texas was disadvantaged. As a thought experiment, and noting that the Texas team sought support from the US government's “Operation Warp Speed” but was rebuffed,^1^ imagine that public funders did get behind this project, conditional on Texas partnering with a major pharmaceutical firm. Such a scenario would not have been entirely different from what transpired in the United Kingdom with Oxford, which was encouraged to find a partner like AstraZeneca, and the subsequent trajectories of the vaccines may have been more similar, with both featuring the sort of multilevel arrangement we described above in the case of Oxford-AstraZeneca. Thus, far from being necessarily and inextricably linked, the licensing approaches and the subsequent outcomes of the Texas and Oxford vaccine programs should be regarded as functions of the innovation ecosystems within which they were operating. Neither of these approaches was inherently better or worse than the other, but in the innovation ecosystems within which they were operating, the Oxford approach achieved a greater global health impact.

Although Oxford's decision to partner with AstraZeneca has been characterized as an abandonment of its commitment to global public health, this characterization is not entirely fair. After all, even if the Oxford approach was not open source,^41^ it was not a standard commercial approach either. As discussed, the Oxford-AstraZeneca agreement included expectations that AstraZeneca would engage in technology transfer to construct a global production network, as well as placing constraints on the subsequent distribution and pricing of vaccine output. In this sense, Oxford's “conditional licensing” strategy that combines commercial and public health considerations can be compared to the “ethical licensing” approach adopted by the Broad Institute of Harvard and the Massachusetts Institute of Technology (MIT). Under this approach, the Institute included in its commercial licenses of its patented CRISPR gene editing technology restrictions against certain objectionable uses of the technology such as tobacco enhancement and species-destroying gene drives.^42^ But while other large universities publicly announced programs to make their technologies available on generous terms during the COVID-19 pandemic, the results of these commitments, if any, have not been announced,^15^ and the Oxford approach appears to have had a greater impact on public health.

Last, we wish to acknowledge that our article does not offer a comprehensive comparison of all aspects of the 2 university-based projects, but rather a focused analysis meant to draw attention to the important role played by active technology transfer. Ultimately, we believe that the comparison of the Texas and Oxford vaccine projects sheds light on how the innovation ecosystem functioned during the COVID-19 pandemic.^43^ The different strategies for vaccine distribution adopted by these leading academic groups suggests that creative thinking and hybrid approaches may be needed to deal effectively with future pandemics and other global health challenges.

## Supplementary Material

qxae012_Supplementary_Data
